# RNA therapy is shining for genetic diseases

**DOI:** 10.1016/j.omtn.2023.102042

**Published:** 2023-10-17

**Authors:** Zhi-Ming Zheng

**Affiliations:** 1 Tumor Virus RNA Biology Section, HIV Dynamics and Replication Program, Center for Cancer Research, National Cancer Institute, National Institutes of Health, Frederick, MD 21702, USA

**Figure 1:**
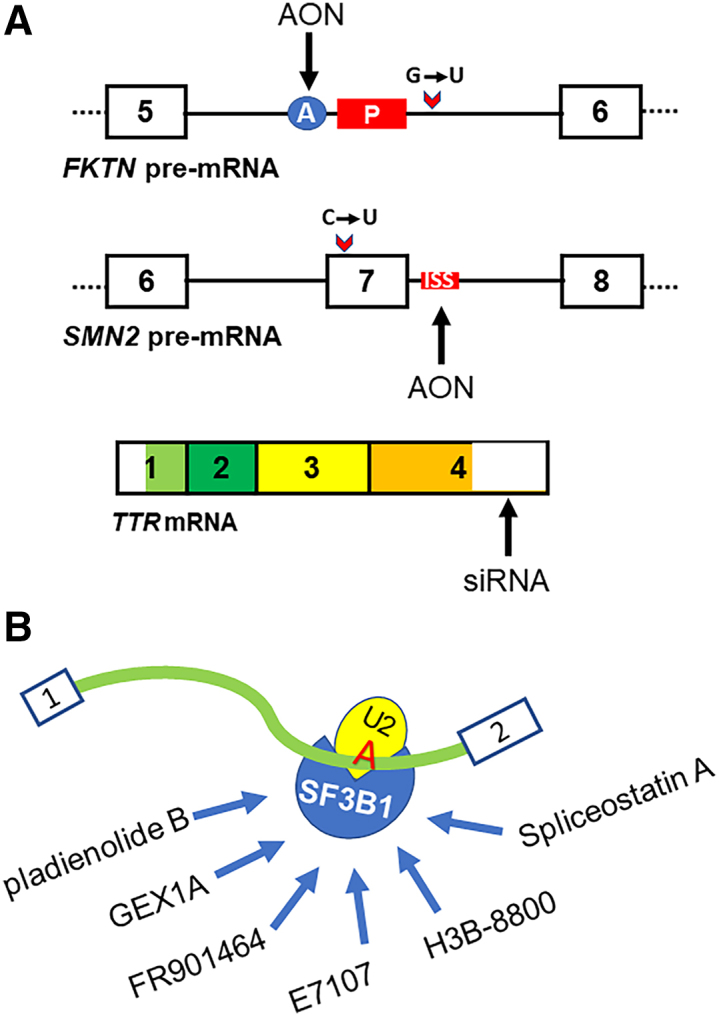
Correction or suppression of aberrant RNA splicing by specific RNA-targeted approaches and SF3B1-targeted splicing modulators (A) AONs or small interfering RNAs (siRNAs) in RNA therapy of genetic diseases. A G-to-U mutation in *FKTN* intron 5 activates a 64-nt pseudo-exon (P) inclusion in *FKTN* mRNA and reduction of glycosylated α-DG in FCMD patients. A BP-AON specifically targeting to the BPS upstream of the pseudo-exon promotes the pseudo-exon exclusion for production of the normal *FKTN* mRNA.^3^ SMN2 gene encoding 9 exons has a C- to-T mutation in exon 7 in SMA patients, which promotes exon 7 skipping in SMN2 pre-mRNA splicing, resulting in a truncated dysfunctional SMN2 protein to cause severe SMA.^4^ The AON-based Nusingersen (Spinraza) targeting an intronic splicing silencer in SMN2 intron 7 prevents the exon 7 skipping and modifies clinical outcome of SMA.^4^^,^^5^ Transthyretin (TTR) transports the thyroid hormone thyroxine and retinol to the liver and its misfolding and aggregation is associated with hereditary TTR amyloidosis. The siRNA-based Patisiran (Onpattro) targeting the 3′ UTR (white box) of both wild-type and mutant TTR mRNAs induces RNA degradation to reduce the production of TTR protein.^6^ Figures are not in scale. (B) SF3B1 facilitates U2 binding to a branchpoint A in BPS to initiate RNA splicing. Small-molecule splicing modulators targeting SF3B1 in regulation of RNA splicing are currently under investigations for possible anticancer therapies and treatment of genetic disorders.

RNA vaccines and RNA therapeutic applications have been two astonishing and fascinating directions in modern biomedical world. RNA vaccines made separately by Pfizer/BioNTech and Moderna are two U.S. Food and Drug Administration (FDA)-approved mRNA vaccines for expressing a transmembrane severe acute respiratory syndrome coronavirus 2 (SARS-CoV-2) spike protein, a viral surface protein interacting with host-cell receptor angiotensin-converting enzyme 2, to induce humoral and cellular immunity against SARS-CoV-2 infection.^1^^,^^2^ Nusinersen, commercially marketed as Spinraza, is an FDA-approved antisense oligonucleotide (AON) drug in 2016 for the intrathecal treatment of spinal muscular atrophy (SMA). Nusinersen prevents skipping of SMN2 exon 7 by AON targeting an intron 7 region to restore normal RNA splicing (Figure 1A) and thus modifies the clinical outcome of SMA.^4^^,^^5^ Patisiran, commercially marketed as Onpattro and approved by FDA in 2018, is a double-stranded small interfering RNA targeting the 3′ UTR of both wild-type and mutant transthyretin mRNAs for intravenous treatment of polyneuropathy in patients with hereditary transthyretin amyloidosis^6^ (Figure 1A). In the September issue of *Molecular Therapy: Nucleic Acids*, Ohara, Hagiwara et al. explored RNA branchpoints as possible potential targets of exon-skipping therapies for treatment of Fukuyama congenital muscular dystrophy (FCMD), which is caused by mutations in the *Fukutin* (FKTN) gene.^3^ Hiroaki, Hagiwara et al. designed RNA branchpoint-targeted AONs (BP-AONs) to block a pseudo-exon inclusion of mutant FKTN RNA (Figure 1A). The authors showed that this BP-AON could restore normal *FKTN* RNA splicing by exclusion of the pseudo-exon in FCMD patient myotubes.^3^

The human genome is made of >3.055 billion base-pairs of nucleotide sequence, with 126,591,489 of single nucleotide variations per individual genome^7^^,^^8^ and encodes approximately 19,969 protein-coding genes for the production of 86,245 RNA transcripts (4.31 transcripts per gene).^7^ Approximate 96% of the protein-coding genes are split genes that are made of introns (intervening sequences) and exons (noncoding or coding segments represented in the mRNA from RNA splicing). Each intron has a 5′ splice site (5′ ss or splicing donor site) and a 3′ ss (splicing acceptor site). Most human and viral introns are GT-AG introns (98.7%) starting with a GT dinucleotide at the intron 5′ end and ending with an AG dinucleotide at the intron 3′ end. A branchpoint sequence (BPS, a mammalian or viral consensus heptamer YNYUR**A**C with branchpoint A underlined) upstream of 3′ ss is separated by a run of 15–40 pyrimidines called a polypyrimidine tract (PPT).^9^ Depending on the sequence context of PPT and branchpoint and the surrounding sequences of the 5′ ss and 3′ ss, an intron could be optimal or suboptimal and may also bear cryptic splice sites and alternative BPS in close proximity to each other or in distance.^10^^,^^11^ Mechanistically, RNA splicing by spliceosome scanning in a 5′ to 3′ direction takes place by small nuclear RNA (snRNA) U1 binding to the intron 5′ ss and snRNA U2 to the BPS accelerated by PPT binding proteins, resulting in recognition of the 3′ ss to trigger the intron splicing and exon-exon ligation.^9^ A precursor mRNA (pre-mRNA) transcribed from a split gene contains the same order of introns and exons in the gene and undergoes RNA splicing before becoming a mRNA for protein translation. However, alternative RNA splicing^9^ due to suboptimal features of the intron or activation of cryptic splice sites in the intron may occur in a tissue- or cell type-specific manner or due to *FKTN* mutations in the case of FCMD and result in production of different isoforms of the mRNA and, thus, of truncated proteins.

FCMD is the most prevalent genetic disease in Japan (also in Korea, China, Greece, Italy, and the United States, etc.) and is caused by mutations and the 3′ UTR insertion of a approximately 3-kb SINE-VNTR-Alu (SVA) retrotransposon in the *FKTN* gene.^12^ FCMD patients with *FKTN* mutations are characterized by a triad of mental retardation, brain malformation, and congenital muscular dystrophy. *FKTN* contains 11 exons spanning over 89,989 bp on Chr9q31–33 and transcribes a 7,456-nt mRNA to encode a 461-aa ribitol-5-phosphate transferase for glycosylation of α-dystroglycan (α-DG),^12^ a cell-surface protein that links the intracellular cytoskeleton and extracellular matrix in muscle, brain, and peripheral nerves. Among 802,674 unique variants in the ClinVar database (https://www.ncbi.nlm.nih.gov/clinvar/), *FKTN* bearing c.647+2084 G>T mutation^13^ in intron 5 creates a new 5′ ss and causes aberrant splicing to include a 64-bp pseudo-exon, which results in a frameshift and premature termination of FKTN protein and consequently, reduces the production of glycosylated α-DG, and causes severe FCMD. To understand how the c.647+2084 G>T mutation could lead a pseudo-exon inclusion and seek for possible treatment of genetic disorders caused by pseudo-exons, Ohara, Hagiwara et al. first established a *FKTN* exon 5-intron 5-exon 6 splicing reporter assay and screened a customized small-molecule compound library.^3^ The authors discovered the enhanced pseudo-exon-skipping by TG003, a CLK inhibitor, and by SF3B1 inhibitors (pladienolide B, GEX1A, and FR901464) (Figure 1B), which exhibited much higher efficiency in exclusion of the *FKTN* pseudo-exon and, thus, restored the normal splicing pattern in *FKTN* mRNA.^3^

Since SF3B1 is a major protein component in U2 snRNP complex for the stable binding of U2 snRNP to the BPS in pre-mRNA splicing^14^^,^^15^ and somatic SF3B1 mutations in myelodysplasia lead to aberrant RNA splicing through reduction of branchpoint fidelity,^16^ these SF3B1 inhibitors, like other SF3B1 modulators spliceostatin A, E7107, and H3B-8800 (Figure 1B),^15^^,^^17^ could globally alter RNA splicing patterns of exons other than the *FKTN* pseudo-exon. A genome-wide map of human splicing BPS in >10,000 genes was recently constructed by targeted RNA sequencing and by split and inverted alignment.^11^ Alternative splicing can be detected at the BPS level both in host and viral gene expression.^10^^,^^11^ Subsequently, Ohara, Hagiwara et al.^3^ mapped the BPS TTCTA**A**C upstream of the pseudo-exon that controls *FKTN* pseudo-exon inclusion and designed several AONs specifically targeting the mapped BPS. The authors^3^ discovered that two AONs targeting the BPS, not the AON targeting the PPT, promoted the pseudo-exon skipping, rectified the aberrant splicing of mutant *FKTN* mRNA, and increased the level of glycosylated α-DG in FCMD patient-derived myoblast myotubes. This held true when this BP-AON strategy applied to rectify the SVA retrotransposal insertion-induced aberrant splicing by SVA exon trapping^18^ in FCMD myoblast myotubes.

Using chemically modified AONs to modulate RNA splicing by targeting splice sites or splicing enhancer/silencer sequences has been extensively explored to reduce aberrant RNA splicing or enhance the normal expression of gene products. An extraordinary, successful example is Nusinersen treatment of children SMA by AON targeting an intronic splicing suppressor in SMN2 intron 7 to modify clinical outcome.^4^^,^^5^ By BP-AON targeting RNA BPS as a possible potential target of exon-skipping therapy for treatment of FCMD, Ohara, Hagiwara et al.^3^ demonstrated for the first time that an intron BPS could be explored as a novel target to specifically modulate aberrant RNA splicing for other mutations-induced genetic diseases and cancers or even some splicing-empowered virus infections.^19^^,^^20^ Although small-molecule splicing modulators, in particular those targeting SF3B1 (Figure 1B), might be useful in the future for anti-cancer therapeutics and antiviral controls,^15^^,^^21^^,^^22^ their broad non-specific effects on RNA splicing, from a therapeutic perspective, might restrict their potential clinical applications. Successful deliveries of RNA vaccines, Spinraza, and Patisiran mediated by lipids or polymers and lipid- or polymer-based nanoparticles^23^ would make this BP-AON approach possible in the development and formulation of a future precision medicine for FCMD and beyond.
